# Cell‐to‐cell heterogeneity emerges as consequence of metabolic cooperation in a synthetic yeast community

**DOI:** 10.1002/biot.201500301

**Published:** 2016-07-05

**Authors:** Kate Campbell, Jakob Vowinckel, Markus Ralser

**Affiliations:** ^1^ Department of Biochemistry and Cambridge Systems Biology CentreUniversity of CambridgeCambridgeUnited Kingdom; ^2^ The Francis Crick Institute, Mill Hill laboratoryLondonUnited Kingdom

**Keywords:** Heat stress, Metabolic cooperation, Oxidative stress, Self‐establishing metabolically cooperating yeast community

## Abstract

Cells that grow together respond heterogeneously to stress even when they are genetically similar. Metabolism, a key determinant of cellular stress tolerance, may be one source of this phenotypic heterogeneity, however, this relationship is largely unclear. We used self‐establishing metabolically cooperating (SeMeCo) yeast communities, in which metabolic cooperation can be followed on the basis of genotype, as a model to dissect the role of metabolic cooperation in single‐cell heterogeneity. Cells within SeMeCo communities showed to be highly heterogeneous in their stress tolerance, while the survival of each cell under heat or oxidative stress, was strongly determined by its metabolic specialization. This heterogeneity emerged for all metabolite exchange interactions studied (histidine, leucine, uracil, and methionine) as well as oxidant (H_2_O_2_, diamide) and heat stress treatments. In contrast, the SeMeCo community collectively showed to be similarly tolerant to stress as wild‐type populations. Moreover, stress heterogeneity did not establish as sole consequence of metabolic genotype (auxotrophic background) of the single cell, but was observed only for cells that cooperated according to their metabolic capacity. We therefore conclude that phenotypic heterogeneity and cell to cell differences in stress tolerance are emergent properties when cells cooperate in metabolism.

**Abbreviations**
CFUs: colony forming units
mtGFP: mitochondrial localized green fluorescent protein
NADPH: nicotinamide adenine dinucleotide phosphate
PPP: pentose phosphate pathway
SeMeCo: self‐establishing metabolically cooperating

## 1 Introduction

Metabolism and the stress response are two highly interdependent processes: metabolism determines the growth rate of cells, provides cofactors for the stress responsive machinery (i.e. nicotinamide adenine dinucleotide phosphate (NADPH) for antioxidant enzymes), and is a source of toxic or oxidizing molecules itself, while the stress response involves metabolic re‐configuration 1, 2, 3, 4. A prime example of the role of metabolism in stress situations is the glycolysis/pentose phosphate pathway (PPP) transition, which acts as a first line antioxidant system conserved from yeast to mammalian cells. Within seconds of contact to oxidative stress, cells temporarily block glycolysis and in parallel increase activity within the PPP 5. This provides immediate protection for the cell due to the increased production of NADPH, which is then available for antioxidant enzymes, such as thioredoxin reductase and glutathione reductase, to regenerate one of the cell's principal antioxidant metabolites, reduced glutathione (GSH) 5, 6, 7, 8. Due to the broad importance of redox cofactors for antioxidant enzymes, many additional metabolic pathways that involve these cofactors also impact cellular stress tolerance, such as the Krebs cycle, the respiratory chain, the methionine, leucine and lysine biosynthetic pathways, the kynurenine pathway important for tryptophan degradation and NAD^+^ synthesis, and the polyamine pathway to name a few 9, 10, 11, 12, 13, 14, 15.

The stress tolerance of a cell community does not necessarily reflect the stress tolerance of its individual cell members however. Even when genetically homogeneous, co‐growing cells establish substantial diversity, with some cells surviving and others dying upon application of the same perturbation 16, 17, 18, 19, 20. Such heterogeneity can be non‐genotypic and, by enabling bet hedging strategies, positively influence the survival of the community in stress situations 21, 22. Phenotypic heterogeneity hence improves population fitness in a fluctuating environment 23. For bacteria, the extreme tolerance to stress for a few cells within a community is classically referred to as persistence, with recent studies showing metabolism to play a critical role in this survival mechanism 24, 25. Furthermore cells that persist against stress treatment themselves, exhibit cell‐to‐cell phenotypic heterogeneity 25. The ability of cells to respond heterogeneously to stress consequently has a major impact on sterilization processes as well as efforts to combat microbial infection 26. From a biotechnology perspective, phenotypic heterogeneity has been considered an exploitable cellular property. For instance, phenotypic heterogeneity has enabled the selection of progressively more stress tolerant cells over time, increasing yeast ethanol tolerance 27, or increasing tolerance to lignocellulose hydrolysates inhibitors and subsequently improving xylose fermentation 28. Phenotypic heterogeneity can therefore improve population fitness in large scale bioreactor processes where environmental fluctuations occur as a result of transient concentration gradients 29. Despite these promising avenues of research, phenotypic variability also gives rise to difficulties in controlling bioprocessing 30. One cause of suboptimal productivity can be the presence of subpopulations exhibiting non‐producer and low‐producer phenotypes as a result of the metabolic state of the cell, such as its stage in the cell cycle or its interaction with other cells in the population 30, 31.

Phenotypic heterogeneity is by no means a speciality of single cell organisms. Chinese hamster ovary (CHO) cells, a commonly used mammalian cell line for therapeutic protein mass production, also exhibit phenotypic heterogeneity. CHO subclones isolated from their parental population exhibit interclonal heterogeneity, with differences in key attributes which may impact biomanufacturing processes 32. Small metazoans such as *Caenorhabditis elegans*, also show to be phenotypically heterogeneous, implying that this cellular phenomenon is a common biological property 33.

Stochasticity or noise in gene expression are discussed as predominant molecular causes of this cellular heterogeneity 34, 35, 36. However, alternative biochemical causes, such as cell‐to‐cell differences in metabolism, have begun to receive significant attention 22, 30, 37, 38. To understand the role of metabolism in this process, it would be desirable to measure the metabolic exchange flux between individual cells and link this information with the phenotypic heterogeneity of the population. However, technical difficulties have dampened these efforts so far 39. Subsequently there is a significant gap in our understanding of what mechanisms are behind cell‐to‐cell metabolic heterogeneity, how they are associated with metabolism and how we may exploit them for clinical intervention, or for improving biotechnological processes.

The use of synthetic biological systems can be beneficial when it is hard to obtain observations under native conditions. We recently developed a synthetic system, termed self‐establishing metabolically cooperating communities (SeMeCos) in which the metabolic role of a cell can be tracked based on its genotype 31. This system starts from a single cell that grows into a progressively heterogeneous community, whose cellular members are increasingly dependent on metabolic cooperation for survival. We designed this system to study cell‐to‐cell metabolite exchange interactions that occur under normal physiological conditions between co‐growing cells in budding yeast. These self‐establishing communities are exploited here to gain insight into the role of metabolic cooperation in cells establishing stress resistance diversity at the single cell level. We report evidence that the metabolic role of a cell, which participates in a cooperating community, is a strong determinant of its survival chance in a given stress situation. Single‐cell heterogeneity in regards to stress tolerance would hence be an emergent property when cells cooperate and specialize in metabolism.

## 2 Materials and methods

### 2.1 Yeast strains, plasmids and growth media

Yeast strains and plasmids have been described previously 31. For microscopy analyses of uracil abundance in SeMeCo populations under stress, BY4741 with prototrophy restored by complementation with p423 (*HIS3*), pRS425 (*LEU2*), pRS411 (*MET15*) and p426‐GPDpr‐mCherry, to express the red fluorescent protein (RFP) in *URA3* cells, was used. To visualize mitochondrial morphology, mitochondrial networks were labelled and analyzed with the dual‐marker plasmid pMitoLoc 40 (Addgene number: 58980) that labels mitochondria with green fluorescent protein (mtGFP) 40, 41.

Yeast was cultivated, if not otherwise indicated, at 30°C in minimal supplemented synthetic media (SM: YNB yeast nitrogen base, Sigma, 6.8 g/L), complete supplemented synthetic media (SC: CSM complete supplement mixture, MP Biomedicals; 0.56 g/L; YNB yeast nitrogen base, Sigma, 6.8 g/L), or rich media (YPD: 1% yeast extract, BactoTM; 2% peptone, BactoTM) with 2% glucose (Sigma) as the carbon source. Media recipes and amino acid compositions were used as previously published 42.

SeMeCo colonies were established as previously described 31. In brief, a founding colony carrying four plasmids that compensate for the genomic deficiency of *HIS3*, *LEU2*, *MET15* and* URA3* of BY4741 43 was grown on minimal media, and re‐diluted and re‐spotted every 48 h for seven days to enable segregation of the plasmids.

### 2.2 Oxidative stress for individual auxotrophs and prototrophs

To compare the respective auxotroph tolerances to oxidants when there is complete media supplementation, prototrophic BY4741 and its single auxotrophy derivatives 42 were pre‐cultured overnight in complete media (SC), a day culture was seeded at approx. 4.0 × 10^6^ cells/mL in SC and cells were collected at mid‐exponential growth phase. Strains were normalized to approx. 1.2 × 10^7^ cells/mL in SC and spotted in 1:5 serial dilutions on SC solid media with H_2_O_2_. Growth was then documented after three days incubation at 30°C.

To test the effect of nutrient supplementation on oxidant tolerance, prototrophic YSBN5 cells were cultured overnight in synthetic minimal (SM) media ± supplementation of histidine (20 mg/L), leucine (60 mg/L), uracil (20 mg/L) and/ or methionine (20 mg/L). Stationary cells were normalized to approx. 1.8 × 10^7^ cells/mL in H_2_O and spotted in 1:5 serial dilutions on SM solid media matching the supplementation of the overnight culture ± H_2_O_2_. Growth was then documented after three days incubation at 30°C.

### 2.3 Oxidative stress and heat shock for colony and metabotypes

To determine oxidant and heat tolerance for yeast strains, cells were pre‐grown for 48 h on SM solid media to establish a giant colony. To determine total population's oxidant tolerance, colonies were re‐suspended in H_2_O and normalized to approx. 3.6 × 10^6^ cells in 200 μL SM and spotted in 1:5 serial dilutions on SM solid media supplemented with either diamide (Sigma) or H_2_O_2_ (Sigma). Growth was then documented after three days incubation at 30°C. To analyze total population heat tolerance, colonies were re‐suspended in H_2_O and diluted to approx. 4.5 × 10^6^ cells in 250 μL SM then subjected to 5 min of heat shock (30, 53 and 55°C) in a water bath. Lag phases were determined from growth curves using a model‐richards fit from the R ‘grofit’ package 44.

To determine percentage cell viability after oxidant stress alongside varying nutrient supplementation, cells were normalized to approx. 2.4 × 10^3^ in H_2_O and plated on SC and drop out solid media (SC without either methionine, leucine, uracil or histidine) ± diamide. Following three days incubation at 30°C, the number of colony forming units (CFUs) were automatically counted using Cell Profiler.

### 2.4 Mitochondrial morphology studies in super resolution

SeMeCo 31 colonies containing the pMitoLoc 40 marker were established for seven days by re‐dilution and spotting once every 48 h, on SM solid media containing 100 µg/mL nourseothricin (NAT; Werner BioAgents) to select for pMitoLoc. Prior to stress tests, cells were spotted and grown for 48 h on SM solid media and 100 µg/mL NAT to establish a giant colony. Colonies were then re‐suspended in H_2_O and diluted to approx. 2.7 × 10^7^ cells in 1.5 mL H_2_O, and treated with H_2_O_2_ for 45 min at 30°C with shaking (750 rpm). Following H_2_O_2_ treatment, yeast cells were collected by centrifugation, washed in PBS and fixed using paraformaldehyde (PFA) solution (4 g/L PFA, 3.6% sucrose) for 20 min. Cells were then centrifuged and re‐suspended in PBS containing 10 µg/mL Calcofluor White (Sigma) and incubated at room temperature for 5 min. Cells were then washed twice with PBS and re‐suspended in 20 µL Vectashield mounting medium (Vector Labs). 1.5 µL of suspension was then applied to poly‐L‐lysine coated microscope slides.

To quantify relative abundances of uracil genotypes within the colony population, with or without H_2_O_2_ treatment, we used conventional wide field fluorescence microscopy on an Olympus IX81 microscope (Deltavision, GE Healthcare) equipped with a 60x 1.42NA PlanApoN oil objective (Olympus). The filter sets used were TRITC for mCherry labelling (555/28 ex, 617/73 em) and DAPI for Calcofluor White (360/40 ex, 457/50 em). Images with a z‐spacing of 200 nm were recorded with a CoolSNAP HQ2 CCD camera. Deconvolution was performed using Softworx software (GE Healthcare).

For mitochondrial morphological analysis of single cells with or without H_2_O_2_ treatment, super‐resolution fluorescence microscopy was carried out using a Deltavision 3D‐SIM OMX system (GE Healthcare) equipped with a 60× 1.4NA oil objective (Olympus), 405 nm (Calcofluor), 488 nm (GFP) and 594 nm (mCherry) laser lines, and the OMX Standard filter set drawer. Images were acquired in structured illumination mode using a z‐spacing of 125 nm, and reconstructed using Softworx software as described in 40. Images were cropped to contain one single cell and subjected to volumetric analysis of their mitochondrial network using Volocity software (Perkin Elmer), or analyzed in ImageJ using the MitoMap plugin 40. For each cell, relative mitochondrial volumes (*V*_s_) were calculated 40, and objects with *V*_s_ < 20% were considered fragmented.

### 2.5 Oxidative stress and heat shock for metabotypes

To investigate oxidant and heat shock tolerance for individual metabotypes, cells were first pre‐grown for 48 h on SM solid media to establish giant colonies. Colonies were then re‐suspended in H_2_O, and samples were normalized to approx. 4.5 × 10^7^ cells/ mL. For oxidative stress treatment, cells were plated at 1:100 000, to isolate individual CFUs, on SC solid media containing either no oxidant, diamide or H_2_O_2_ in the sublethal to lethal concentration range. CFUs on plates containing oxidant concentrations immediately less than plates with lethal oxidant concentration were picked for replica plating to determine cell metabotypes. To elucidate individual metabo type heat tolerances, normalized cells were exposed to heat shock at 60°C at a range of incubation times and then plated at a 1:100 000 dilution on SC solid media. Viable cells at heat shock incubation time immediately less than the lethal incubation time were picked for replica plating to determine cell metabotype.

## 3 Results

### 3.1 Cooperating yeast communities are composed of heterogeneously stress resistant cells

**Figure 1:**
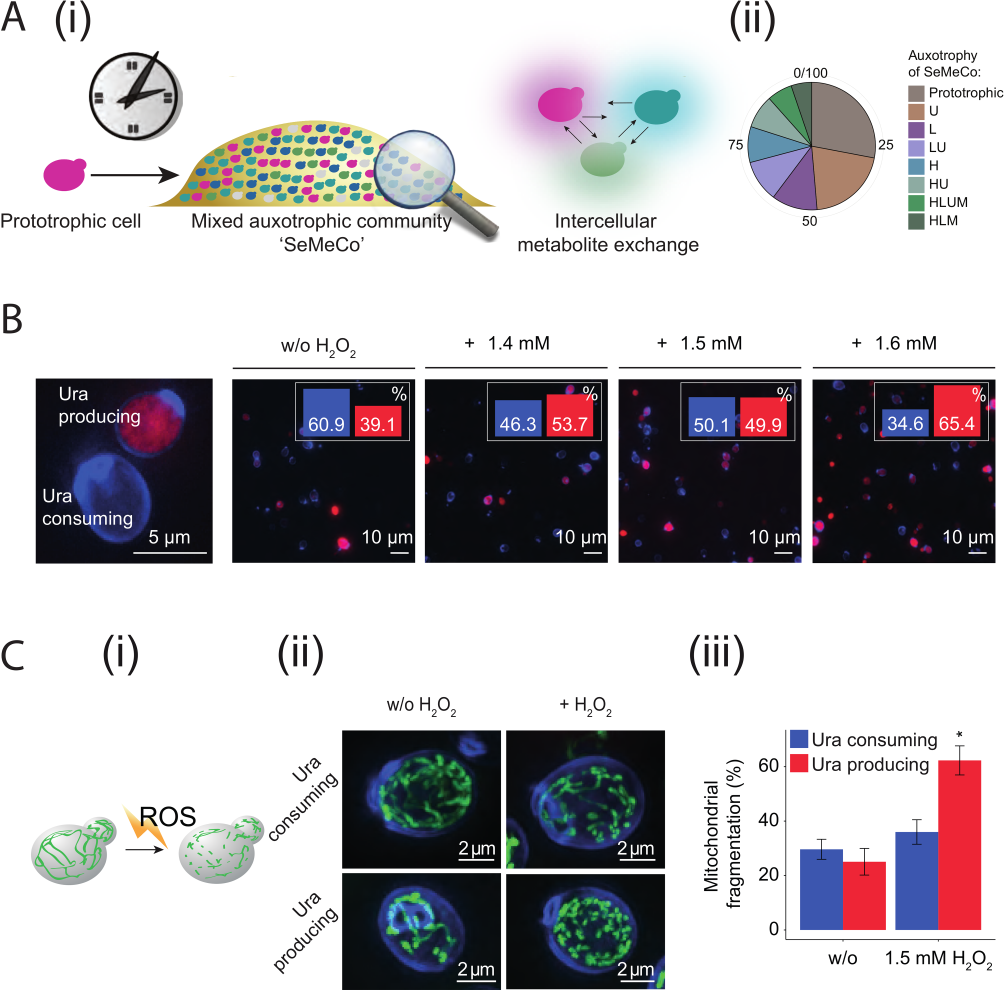
Metabolically cooperating synthetic yeast communities are composed of phenotypically heterogeneous cells. (**A**) (**i**) Scheme of a self‐establishing metabolically cooperating community in *Saccharomyces cerevisiae* (SeMeCo), establishing from an initially prototrophic founder over time. While the colony maintains growth on minimal media, single cells within SeMeco progressively lose prototrophy by the stochastic loss of complementing marker plasmids, and continue growth only if they can share the essential metabolites. (**ii**) Auxotrophic cell types (metabotypes) present in a SeMeCo community established using *HIS3*, *LEU2*, *MET15* and *URA3* as markers. Formed of a total of 16 genotypes, only eight metabotypes are successful cooperators and form the community. Eight rare metabotypes (unsuccessful cooperators), in total contributing to less than <5% of population, are not represented. (**B**) Metabolic cooperation diverges stress tolerance of uracil producing and consuming cells. Uracil consumers within a SeMeCo grown on minimal media, are depleted upon H_2_O_2_ treatment, as determined by fluorescence microscopy, *n* = 1492. Bar charts inset: percentage of uracil consuming (blue, Calcofluor White (CFW) cell wall stained) and producing cells (red, expressing mCherry linked to *URA3*). (**C**) Mitochondrial morphology upon H_2_O_2_ treatment distinguishes uracil consuming and producing cells within a SeMeCo community grown on minimal media. (**i**) Scheme of mitochondrial network disintegration (fission) following exposure to H_2_O_2_. (**ii**) Super‐resolution fluorescence microscopy image of the fluorescent labelled mitochondrial network, using pMitoLoc, for both uracil consuming and uracil producing cells within SeMeCo. Cell walls are stained with CFW (blue). (**iii**) Exposure to H_2_O_2_ leads to mitochondrial fission in the uracil producing, but not in uracil consuming metabotypes (*n* > 16, error bars = SEM, * *p* < 0.01).

SeMeCo communities start with a metabolically competent (prototrophic) single cell that has several metabolic deficiencies (auxotrophies) complemented by plasmids containing the metabolic genes that are deleted in its genome. When the SeMeCo founder cell grows into a community, these plasmids are stochastically lost at a rate of ~2–4% per cell division so that, over time, the number of auxotrophic cells in the community increases. The resultant communities become progressively metabolically heterogeneous until a minimum number of metabolite producing cells, required to supply the community with the metabolites, is reached. SeMeCo's that share histidine, leucine, uracil and methionine are fully viable, and adopt metabolic capacities similar to wild‐type cell communities, and survive on the basis of nutrient exchange occurring between up to 16 auxotrophic genotypes (metabotypes); (Fig. 1A) 31. The wild type‐like growth properties of SeMeCos allowed us to conclude that sharing of histidine, leucine, uracil and methionine metabolites is a natural property of yeast colonial growth 31. In this manuscript, we exploit the fact that once segregation of a SeMeCo has progressed, one can deduce from the auxotrophic genotype of each single cell which of the four metabolites it produces for the community, and which metabolites it consumes from the pool of shared goods 31.

As metabolism is a key factor in stress tolerance, we questioned whether the exchange of histidine, leucine, uracil and methionine affects the survival chances of the cooperating cells in stress situations. We started by comparing the H_2_O_2_ tolerance of uracil consuming and uracil producing cells – when growing cooperatively in media lacking uracil – as these cell types were the largest complementary group of metabolite consuming and producing cells (Fig. 1A, ii). Uracil consuming cells were more sensitive to H_2_O_2_ than uracil producing cells: The higher the sublethal H_2_O_2_ concentration, the fewer uracil consuming cells were found to constitute SeMeCo (Fig. 1B). We corroborated a difference between uracil producers and uracil consumers by exploiting a morphological feature of mitochondrial networks that allowed us to compare the response of single cells during H_2_O_2 _treatment. Under normal growth conditions, mitochondria fuse to form large tubular networks, however, when cells are exposed to H_2_O_2_ they undergo fission into fragmented mitochondrial units (Fig. 1C, i and ii) 2, 45. We made use of a recently developed strategy and mathematical framework (MitoLoc) to numerically express such changes in single cells 40. The method makes use of mitochondrial localized GFP (mtGFP) 41 pictured on an OMX super resolution microscope 46 and calculates morphological features upon automated 3D reconstruction 40. In order to apply the method, a SeMeCo community carrying the mitochondrial marker pMitoLoc was re‐established. Without oxidant, the mitochondrial network of SeMeCo's uracil producing and consuming cells showed a similar degree of fragmentation (Fig. 1C, iii). At an increased H_2_O_2_ concentration, mitochondrial fission occurred in uracil producing but not in uracil consuming cells (Fig. 1C, iii). The activation of mitochondrial fission, an integral part of the H_2_O_2_ response, was hence dependent on whether the individual cell in SeMeCo was contributing or consuming uracil from the pool of shared goods.

**Figure 2:**
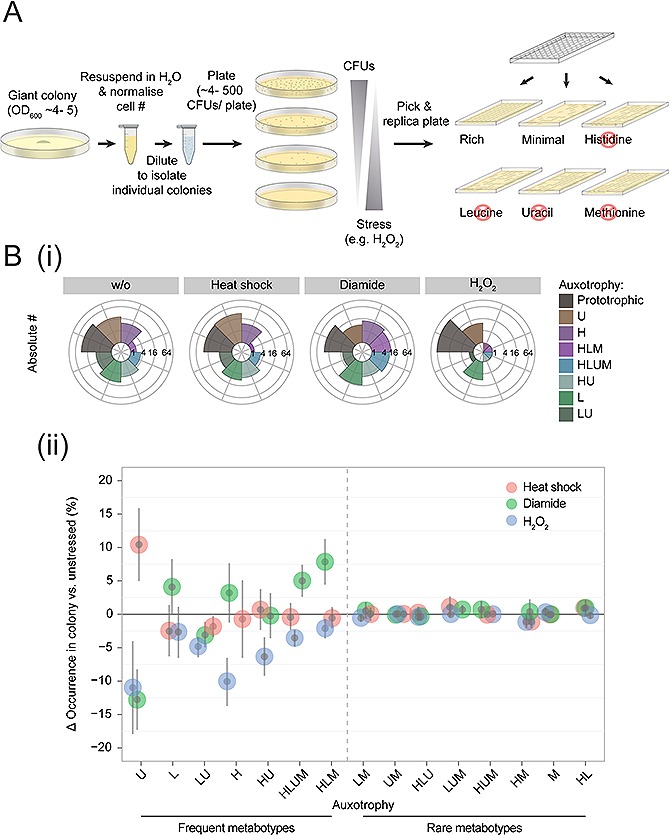
Phenotypic diversity establishes for multiple stress conditions, but only for cooperating metabotypes. (**A**) Scheme for determining individual metabotype's stress tolerance in SeMeCo giant colonies, via replica plating. (**B**) (**i**) The metabotype composition of SeMeCo changes upon to exposure to heat (60°C for 50 seconds, *n* = 185), to diamide (1.2 mM, *n* = 282) or to H_2_O_2_ (0.2 mM, *n* = 270 genotyped cells). (**ii**) Relative change to unchallenged cells (from x‐axis when *y* = 0) of individual metabotype's survival upon exposure of SeMeCo to heat, diamide or H_2_O_2_, *n* = 1295 genotyped cells, error bars = ± standard deviation. Metabotypes are sorted (from left to right), according to their frequency in SeMeCo before the stress treatment 31. Diversification is detected only for the frequent metabotypes.

The combinatorial loss of *HIS3*, *LEU2*, *MET15* or *URA3* results in 16 different metabotypes. Not all of these genotypes are however capable of cooperating in SeMeCo, with only eight of the 16 genotypes demonstrating efficient cooperation in the community 31 (Fig. 1A, ii). The difference between the eight frequent, and eight rare genotypes was exploited to distinguish whether the stress response heterogeneity established as a consequence of the auxotrophic marker the cells contain, or as consequence of successful metabolic cooperation. In the former case, all 16 metabotypes would be different in stress tolerance and in the latter case, differences would only occur between the eight successfully cooperating metabotypes. We monitored the survival of all 16 metabotypes following exposure to H_2_O_2_, the thiol oxidizing compound diamide, or to heat stress, by using replica plating, and studied differences between rare and frequent metabotypes (Fig. 2A). The process of replica plating was preferred over alternative methods such as FACS, as it enables (i) the identification of all 16 genotypes, (ii) the detection of cell survival, and finally (iii), whether cells retain colony forming capacity. All stress treatments affected the auxotrophic composition of SeMeCo (Fig. 2B, i) and had substantially diverse effects on the eight frequent metabotypes (Fig. 2B, ii). For example, consistent with the microscopy results (Fig. 1), the most frequent cell type (consuming uracil), was depleted from SeMeCo upon H_2_O_2_ as well upon diamide exposure, however, showed increased persistence upon heat stress (Fig. 2B, ii). The second most frequent metabotype (consuming leucine) instead was heat and H_2_O_2_ sensitive, but resistant to diamide, while the third most frequent metabotype (consuming leucine and uracil) was sensitive to all three conditions (Fig. 2B, ii). Importantly, none of the frequent metabotypes showed to be consistently stress resistant against all three stress conditions. This shows that the survival of metabolically specialized cells within the synthetic community is specific to a given stress situation.

Remarkably, no such diversification was observed for the eight rare metabotypes (unsuccessful cooperators), despite these cells being composed of the same four auxotrophic markers (Fig. 2B, ii). A limitation in the analysis of the rare metabotypes is certainly that their collective total remains below 5% of the total population (heat: *n* = 6/185, diamine: *n* = 14/282 and H_2_O_2_: *n* = 0/270, versus no stress: *n* = 12/557 genotyped cells), for which reason stress sensitive phenotypes may be missed. These results do however confirm that none of the rare metabotypes were resistant to any of the tested stress conditions. In other words, the oxidant and heat resistance of a cell within SeMeCo did not simply correlate with the four auxotrophic marker mutations; heterogeneity in stress resistance was only observed for the metabotypes that successfully cooperated within the community.

### 3.2 Auxotrophic genotype affects stress resistance indirectly

**Figure 3:**
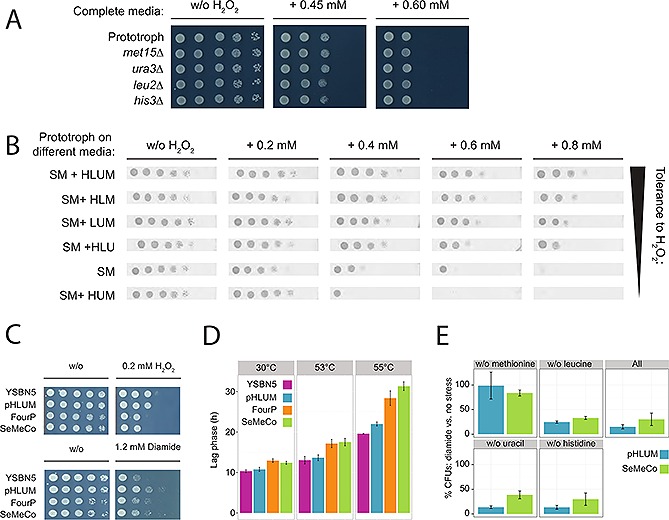
On the community level, SeMeCo and wild‐type colonies possess similar stress tolerances. (**A**) Auxotrophic cells similarly tolerate H_2_O_2_ when fully supplemented. Spot‐testing shows H_2_O_2_ tolerance of methionine (*met15*Δ), uracil (*ura3*Δ), leucine (*leu2*Δ), and histidine (*his3*Δ1) auxotrophs is no different to that of prototrophic cells when cells are grown on synthetic complete (SC) agar media (*n* = 3). (**B**) Prototrophs display a heterogeneous H_2_O_2_ tolerance when media supplementation of histidine, leucine uracil and methionine is varied. The color of the spot test images has been inverted, to facilitate visualization of cell survival across the different plates (*n* = 3). (**C**) Oxidant tolerance of a genomically prototrophic BY4741 43 derivative (YSBN5 53), BY4741 rendered prototrophic with the pHLUM plasmid (pHLUM 42), BY4741 rendered prototrophic with the same plasmids as used to construct SeMeCo (FourP) and SeMeCo 31, as determined with a spotting assay on minimal (SM) solid media containing H2O2 (upper panel) and diamide (lower panel). (**D**) Heat tolerance of YSBN5, pHLUM, FourP and SeMeCo colonies as determined by measuring time to resume growth (lag phase) after 5 min exposure to 30, 53 and 55°C. *n* = 3, error bars = ± standard deviation. (**E**) Oxidant tolerance of single cells constituting pHLUM and SeMeCo colonies, when plated on media with varying amino acid supplementation. Survival is determined as percentage colony forming units (CFUs) that establish on diamide containing plates versus no oxidant being present.

The clear difference between the frequent and rare metabotypes in stress heterogeneity, despite containing the same auxotrophic markers, suggests that the auxotrophic background influences stress tolerance depending on the cells, nutrient uptake profile, but not according to the presence or absence of the marker genes per se. To test this, we exploited the observation that supplemented yeast cells, even when able to synthesize histidine, leucine, uracil, and methionine (genetically prototrophic), take up these four metabolites. We reported previously that uptake by prototrophs occurs at a rate comparable to uptake by auxotrophic cells unable to synthesize these four metabolites, implying that uptake fully meets cellular demands 31, 47. Assayed by spot‐testing on synthetic complete (SC) media, supplemented with the oxidant hydrogen peroxide (H_2_O_2_), all histidine, leucine, uracil and methionine auxotrophs displayed a similar H_2_O_2_ tolerance compared to the prototrophic strain (Fig. 3A). This confirms that these strains, despite their different auxotrophic backgrounds, will have largely similar stress tolerances as long as they have access to the four metabolites.

Conversely, we then tested the H_2_O_2_ tolerance of YSBN5 wild‐type yeast upon different supplementation of histidine, leucine, uracil and methionine. Unlike the homogeneous stress tolerance of the auxotrophs when similarly supplemented, the differently supplemented prototrophs showed heterogeneous resistance (Fig. 3B). The difference in stress tolerance for the auxotrophs thus predominantly originates from their different metabolic activity in the sense of either taking up or self‐synthesizing a given metabolite, but not directly from the absence or presence of the auxotrophic marker gene.

### 3.3 On the community level, the heterogeneously composed SeMeCos behave similarly to wild‐type communities

Not only the individual cells in SeMeCos, but also single cells in yeast wild‐type communities are heterogeneous in their stress resistance 22, 48. Considering cooperative metabolite exchange as an inherent property of yeast colonial growth 31, we thus questioned to what extent the heterogeneously composed SeMeCo communities did, or did not, behave like typical yeast communities in stress tolerance. This would indicate that the stress heterogeneity on the single cell level, emerges as a result of basic yeast metabolic properties, and is not an artificial property of the SeMeCo system. First, spot tests were used to assess the resistance of SeMeCos in comparison to wild‐type yeast colonial communities to the hydroperoxide H_2_O_2_ or the thiol oxidizing compound diamide (Fig. 3C), while growth following heat exposure, was used to assess temperature resistance (Fig. 3D). The tolerance of SeMeCo against both oxidants did not differ significantly to at least one prototrophic colony (Fig. 3C). Heat resistance instead was normal at 53°C but slightly impaired at 55°C for the SeMeCo cells (Fig. 3D). At least to oxidant exposure, SeMeCos were similarly resistant as wild‐type communities. We therefore questioned to what extent this phenotype is reflected on the single cell level. We found largely similar survival numbers, or a slight better performance of the SeMeCo community in some conditions, of individual cells in stressed SeMeCo and wild‐type colonies as determined by measuring colony‐forming capacity (CFUs) upon oxidant exposure (Fig. 3E). Interestingly, wild‐type and SeMeCo communities reacted similarly to each other, and stronger, upon a change in uracil, histidine, leucine and methionine supplementation. Predominant effects were obtained when comparing media with and without methionine, shown to modulate stress resistance over the pentose phosphate pathway 15. Therefore, despite comparing a native yeast community with a SeMeCo community, where metabolic heterogeneity is synthetically tracked in a mostly auxotrophically composed and heterogeneous community, both populations possessed similar survival chances. This result implies that the metabolism‐dependency of cellular heterogeneity is a native property of yeast communities.

## 4 Discussion

When cells co‐grow in proximity, they exchange an array of metabolites. For example, cells within *Saccharomyces cerevisiae* colonies are surrounded by an intra‐colony space rich in metabolites (‘the intra‐colony exometabolome’), containing amino acids and nucleotides. As these metabolites are exchanged at growth relevant quantities, this exometabolome can be exploited by cells to specialize in metabolism 31. Indeed, for several metabolites including histidine, leucine, uracil or methionine, the yeast cells possess an uptake over self‐synthesis preference, so that they readily exploit an available exometabolome to support their metabolic needs 31, 47. As metabolism has a strong and well‐established biochemical influence on oxidative and heat stress resistance 5, 15, 49, 50, it hence seems likely that such metabolic specialization is implicated in single‐cell heterogeneity as observed in stress situations 17, 22, 51. Due to the lack of enabling technologies for tracking metabolite exchange fluxes between single cells, this relationship between metabolism and phenotypic heterogeneity has remained, so far, largely unexplored.

We used self‐establishing communities as a synthetic system where the metabolic function of a single cell can be identified on the basis of its auxotrophic background, to study stress heterogeneity in yeast communities 31. While one cannot directly prove with the SeMeCo system whether stress heterogeneity in normal yeast colonies is a consequence of metabolite exchange, it can be demonstrated that when cells cooperate in metabolism, stress heterogeneity emerges as a consequence of metabolic specialization. Importantly, we could distinguish between the phenotypic heterogeneity caused by the auxotrophic marker genes, and that of the metabolite exchange interactions within SeMeCo. We find the latter to be essential for heterogeneity to establish. Further, wild‐type yeast communities and SeMeCos, whilst heterogeneously composed, were shown to largely corresponded to one another on the colony and single cell survival level, implying that stress heterogeneity that establishes as a consequence of metabolic specialization is a normal property of communal cell growth.

Metabolic exchange activity hence adds to noise in gene expression or somatic mutations 34, 35, 52 as a cause of phenotypic heterogeneity at the single cell level. This finding is important for biotechnology, as cooperation may be targeted to optimize metabolic engineering strategies. By targeting specific metabolic pathways and feedback mechanisms involved in cell‐to‐cell cooperation, it may be possible to enhance cell viability, titer and productivity. Furthermore, these findings could provide a new perspective towards medical research concerning anti‐cancer or antibiotic therapies, where persisting cells compromise therapy success. While both noise in gene expression and somatic mutations are difficult to be targeted pharmacologically, cellular heterogeneity that emerges as a consequence of metabolic cooperativity could be prevented with intelligently designed metabolic inhibitors.
